# Macular edema with serous retinal detachment post-phacoemulsification followed by spectral domain optical coherence tomography: a report of two cases

**DOI:** 10.1186/s13104-015-1639-1

**Published:** 2015-11-04

**Authors:** Hui Xiao, Xing Liu, Xinxing Guo

**Affiliations:** State Key Laboratory of Ophthalmology, Zhongshan Ophthalmic Center, Sun Yat-sun University, Xianlie South Road No 54, Guangzhou, 510060 China

**Keywords:** Macular edema, Retinal detachment, Cefuroxime, Phacoemulsification

## Abstract

**Background:**

Macular edema and detachment at the first day after an uneventful cataract surgery is very rare, and has been reported previously with the use of high concentrations of intra-cameral cefuroxime. However, we hereby reported two cases of macular edema with extensive serous retinal detachment the first day after an uneventful phacoemulsification with intra-cameral injection of a standard dose of cefuroxime during the procedure.

**Case presentation:**

A 68-year-old female and a 63-year-old male without any special history both underwent an uneventful phacoemulsification surgery and 1 mg/0.1 ml of cefuroxime solution was injected into the anterior chamber at the end of the procedure. Macular edema with extensive serous retinal detachment around macula and optic disc area were observed the first day after surgery. Without surgical intervention, a quick recovery of the macular edema and retinal detachment was observed by spectral domain optical coherence tomography 1 week later in both cases.

**Conclusion:**

We presume that the retina injury in the two cases may be attributed to cefuroxime toxicity even under a use of a standard dose. But the retinal damages are restorable and routine anti-inflammatory treatment is enough.

## Background

Macular edema is one of the most common complications after cataract surgery that causes unfavorable visual outcomes and usually occurs in the surgical eye 4–16 weeks after the procedure [1, 2]. Acute macular edema with retinal detachment after cataract surgery is very rare, and has been reported previously with the use of high concentrations of intra-cameral cefuroxime [3, 4]. Cefuroxime is commonly used during phacoemulsification procedure [5] and has been proved to be safe with a standard dose previously [6–8]. However, we hereby report two cases of macular edema with extensive serous retinal detachment that was immediately detected by spectral domain optical coherence tomography (SD-OCT) the first day after an uneventful phacoemulsification with intra-cameral injection of standard doses of cefuroxime during the procedure. We presume that the retina injury in the two cases may be attributed to cefuroxime toxicity even under a use of a standard dose.

## Case presentation

### Case 1

A 68-year-old female had an uncomplicated phacoemulsification surgery with folded in-the-bag intraocular lens (IOL) implantation in her left eye. Her systemic and ophthalmic histories were unremarkable. No diabetic, uveitis or any other remarkable retinal history was found prior to the surgery. Preoperatively, the refractive errors of her right and left eyes were −4.0 and −4.5 diopters (D), with axial lengths of 24.12 and 24.37 mm, respectively. The best-corrected visual acuity was 20/33 in the right and 20/40 in the left eye. The patient’s anterior segment and fundus were normal in both eyes, as revealed by regular examination. The surgery was performed using an Infiniti phacoemulsification unit (Alcon, Inc.). The nucleus chopping time was 5.8 s, and the average power was 6.8 %. A +18 D folded IOL (Acrysof SN60AT Alcon, Inc) was implanted in-the-bag. The surgery was completed without complications. At the end of the procedure, 1 mg/0.1 ml of cefuroxime solution was injected into the anterior chamber. We perform our dilution in the operating room: the nurse takes 750 mg of preservative-free vial cefuroxime and adds 7.5 ml of balanced salt solution (BSS), and then, the surgeon takes 0.1 ml of this first solution and adds another 0.9 ml of BSS to obtain the second solution. 0.1 ml of the second solution is finally injected in the anterior chamber of the patient. The patient is therefore supposed to receive 0.1 ml of 10 mg/ml solution of intra-cameral cefuroxime. The total length of the surgical time was approximately 10 min.

**Fig. 1:**
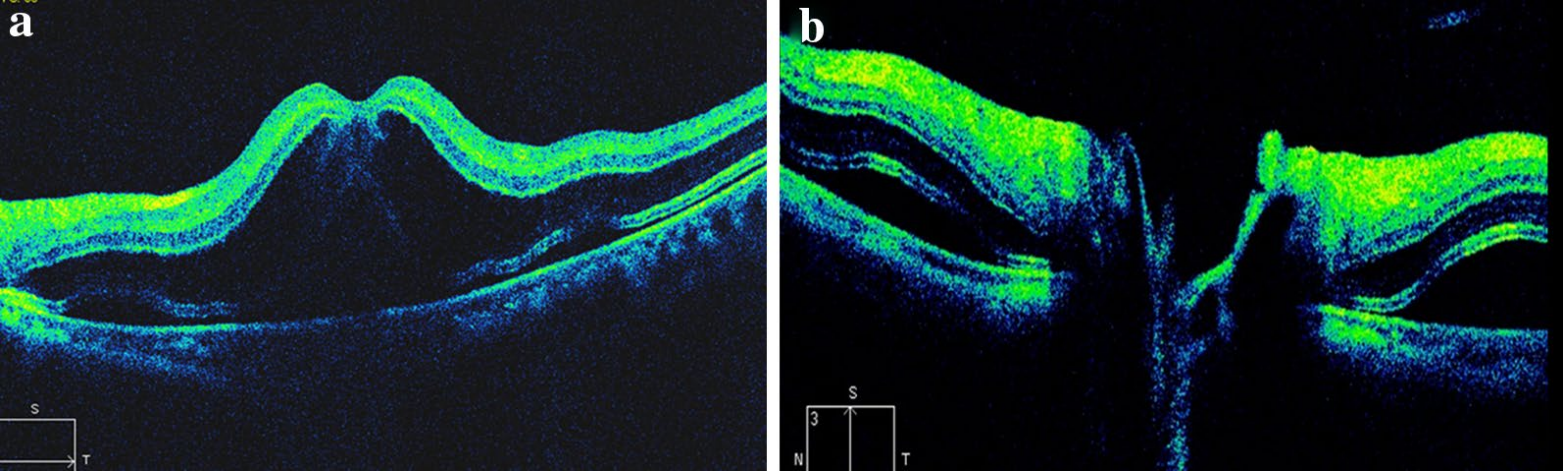
Image of spectral domain optical coherence tomography (SD-OCT) on one day post-operation (case 1). The SD-OCT image demonstrated macular edema most manifested at the outer nuclear layer and shallow serous retinal detachment around the macula (**a**) and optic disc area (**b**)

**Fig. 2:**
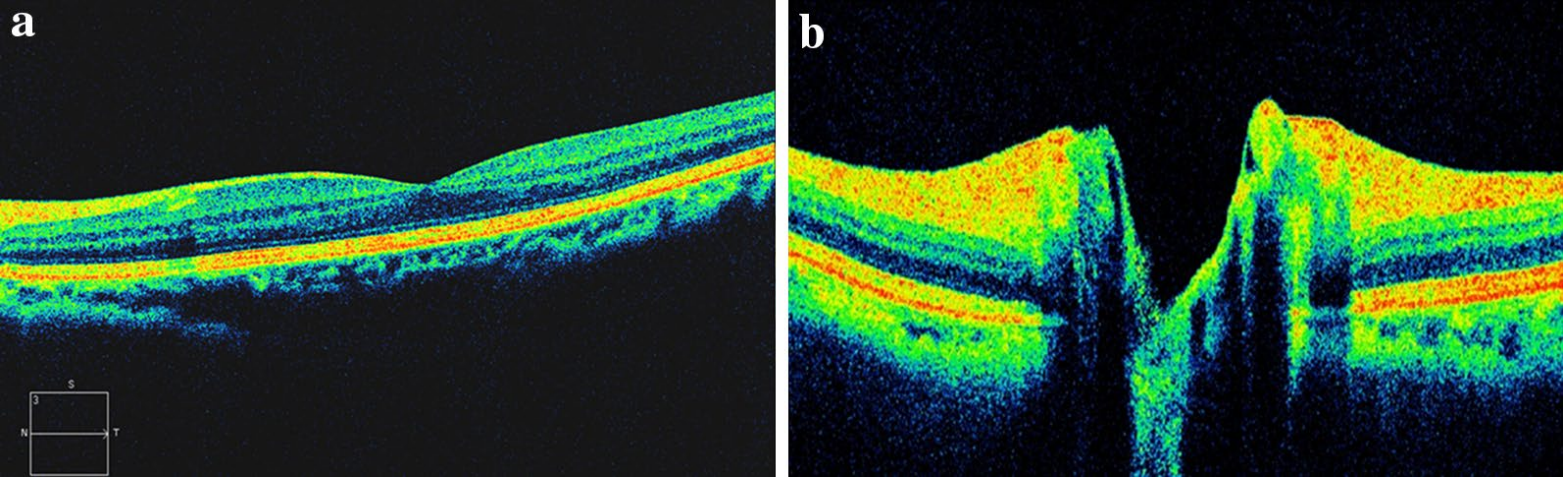
SD-OCT images of case 1 One week post-operation. Macular edema and subretinal fluid was absorbed and the central foveal thickness resumed to normal (**a**). The subretinal fluid was also absorbed around optic disc (**b**)

The first day after the operation, the visual acuity in her left eye was 20/200. There were no signs of remarkable inflammation or abnormality in the anterior segment, as well as in the vitreous. Fundus examination showed no foveal reflection in the macula. Diffuse retinal edema affected most of the posterior pole. Retinal wrinkles were found around the macula and disc area. No significant abnormality was found in the peripheral retina. SD-OCT (Carl Zeiss Meditec, Dublin, CA, USA) scanning was immediately performed and showed macular edema, especially at the outer nuclear layer, with extensive shallow serous retinal detachment around macula and optic disc area (Fig. 1). The retinal thickness of the fovea was 750 μm. No significant abnormality was found in the choroids and the subfoveal choroidal thickness was 350 μm. Vitreomacular traction was not found in the SD-OCT image. Topical dexamethasone 0.1 %/tobramycin 0.3 % (Tobradex^®^) eye drops and pranoprofen (Senju Pharmaceutical Co. Ltd) were prescribed four times a day. After 1 week of treatment, the patient’s vision in her left eye had improved to 20/20. The macular retina was scanned with the same area by SD-OCT and the image showed that the retinal thickness of the fovea returned within a normal range (194 μm), and the subfoveal choroidal thickness seemed not changed a lot (about 347 μm). The macular edema and subretinal fluid were absorbed completely. The integrated ellipsoid zone was preserved in the outer retina (Fig. 2). No recurrence of macular edema or retinal detachment was noted at the last follow-up (4 months post-operative).

### Case 2

A 63-year-old male underwent an uneventful phacoemulsification surgery with folded in-the-bag IOL implantation in the left eye. The systemic and ophthalmic histories were unremarkable. Preoperatively, the refractive error of his left eye was −2.75 D with an axial length of 23.93 mm. The best corrected visual acuity was 20/200. The right eye had IOL implanted 1 year ago with good visual acuity of 20/20. Findings of anterior segment and fundus examination were normal in both eyes. The phacoemulsification surgery was performed by the same doctor as in case 1. The nucleus chopping time was 32 s and the average power was 16.8 %. A +20.0 D folded IOL (Acrysof SN60AT Alcon, Inc) was implanted in-the-bag. The surgery was completed without complications. At the end of the procedure, 1 mg/0.1 ml of cefuroxime solution (diluted as case 1) was also injected into the anterior chamber. The total length of the surgical time was about 12 min.

**Fig. 3:**
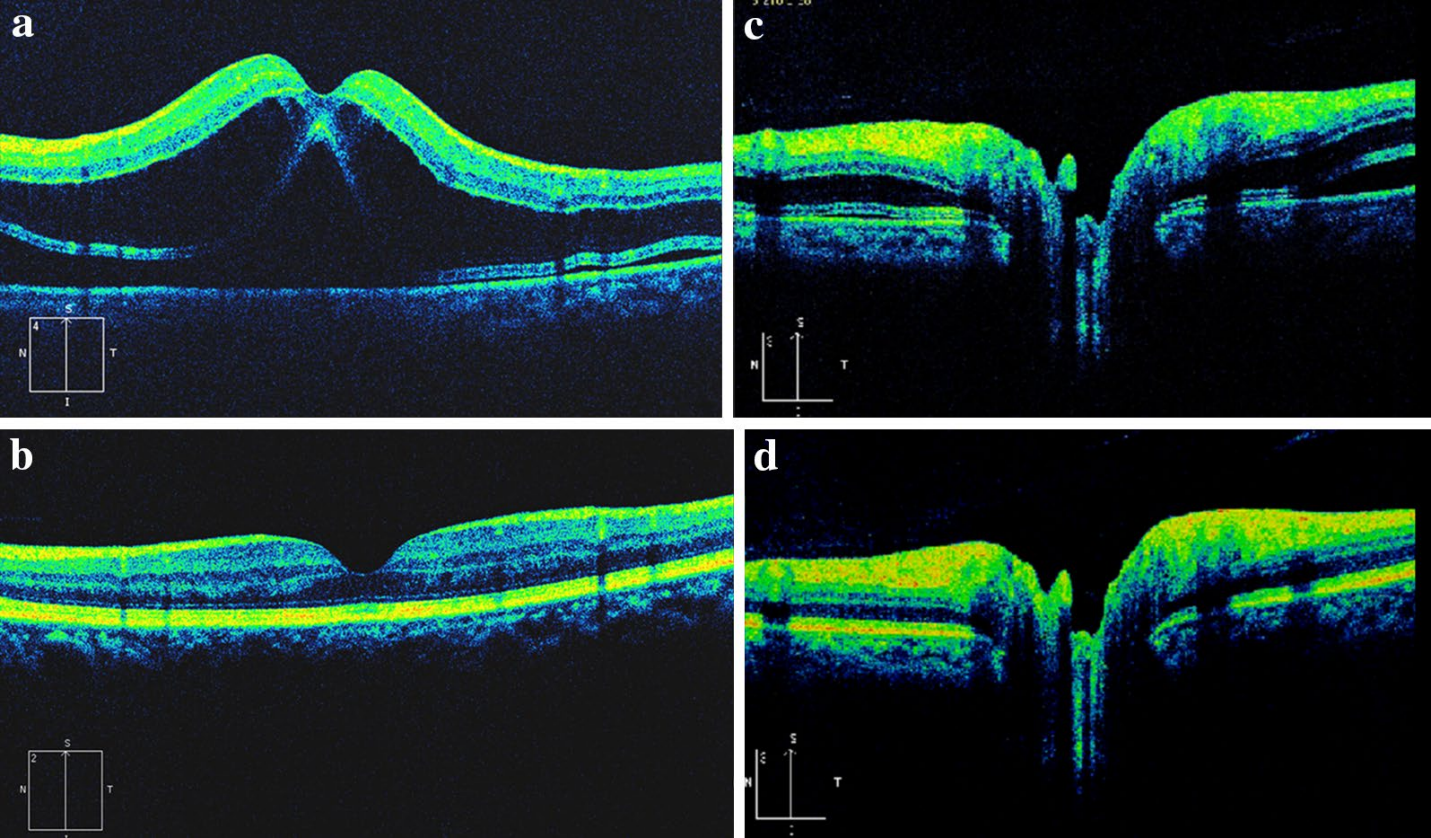
SD-OCT images of case 2. The SD-OCT image demonstrated macular edema most manifested at the outer nuclear layer and extensive shallow serous retinal detachment around the macula (**a**) and optic disc area (**c**) the first day after surgery. Macular edema and subretinal fluid was absorbed and the central foveal thickness resumed to normal at 1 week post-operation (**b**). The subretinal fluid was also absorbed around optic nerve head at 1 week post-operation (**d**)

The first day after the operation, the visual acuity in his left eye was finger count. No remarkable inflammations or abnormalities were found in the anterior segment and vitreous by slit-lamp examination. Fundus manifestation was similar to case 1. SD-OCT image also showed the same macular edema as case 1 (Fig. 3a). The retina thickness of the fovea was 794 μm. No significant abnormality was found in the choroids. The same drugs as in case 1 were adopted four times a day. After 1 week treatment, the patient’s visual acuity improved to 20/20. SD-OCT revealed macular edema and subretinal fluid were absorbed completely. The integrated ellipsoid zone was preserved in the outer retina (Fig. 3b). The retinal thickness of the fovea returned within a normal range (174 μm). No recurrence of macular edema or retinal detachment was noticed until the last follow-up (3 months after surgery).

## Conclusions

With modern cataract surgical techniques, the incidence of post-surgical cystoid macular edema (CME) has decreased to 0.1–2.35 % [9]. Several mechanisms may contribute to such macular edema, including the effects of vitreoretinal traction, light damage, production of prostaglandins and intraoperative complications [1, 10]. The rate of macular edema after cataract surgery is increased in the presence of diabetic retinopathy and uveitis [11, 12]. However, the two cases were notable for its unremarkable retinopathy and lack of history of diabetes or uveitis.

Jurecka et al. [13] found a positive statistical correlation between the real phacoemulsification time and the increase in macular retinal thickness after surgery. In the present cases, the real phacoemulsification time was not long, and the average power was low.

Cefuroxime toxicity may be one of the cause of macular edema and detachment. The recommended dose of intra-cameral cefuroxime injection is 0.1 ml of 10.0 mg/ml solution. The fact that excessive cefuroxime solution injections into the anterior chamber can cause early serous macular detachment and edema has been reported previously [3, 4]. The reported dose has been varied from 20 to 50 mg/ml. However, recently, Kontos et al. [14] reported a case with acute serous macular detachment and macular edema after a standard dose of subconjunctival cefuroxime injection in the phacoemulsification. Faure et al. [15] even reported a case occurred retinal toxicity the second day after surgery with a standard dose of intra-cameral cefuroxime injection in France. In the present two cases in China, early macular edema and extensive retinal detachment were found immediately the first day after surgery with a standard dose of intra-cameral cefuroxime injection at the end of the phacoemulsification. The visual loss was earlier in the present two cases than that of Faure et al. report. Though the visual loss time after surgery had little difference, the manifestations of these cases were similar. The interval time between the present two cases was about one month. No abnormality was found during the drug dilution process. Thus, we presume that the retina injury in the two cases may be also attributed to cefuroxime toxicity even under a use of a standard dose.

In these two cases, the location of the edema was unusual: Typically retinal edema was located in the outer plexiform layer. However, in these cases the outer plexiform layer appeared to be spared and the outer nuclear layer had large edema. There was extensive subretinal fluid without debris. These OCT characteristics were similar to the manifestation that has been identified in OCT of the retinal toxicity caused by excessive cefuroxime solution injections [3, 4], and might provide a marker for cefuroxime toxicity. The mechanism of this pattern of edema is unclear. The electroretinogram (ERG) results of animal experiments [16] and human clinical observation [15] prompted cefuroxime was toxic to retina, and may effect the Müller cell function. Previous study [3, 4] reported that fluorescein angiograms (FA) showed diffuse leakage without abnormal retinal perfusion in cefuroxime toxic eyes and indicated that the blood–retinal barrier at the retinal pigment epithelium (RPE) may be disrupted. It is a limitation that the FA was not obtained in the present two cases, but the SD-OCT images may suggest that the primary lesions were localized at the outer retinal and RPE.

Clear vitreous haze has been reported in ocular toxicity after intra-cameral injection of very high doses of cefuroxime during cataract surgery [4]. But, no sign of remarkable inflammation in the vitreous was found in the present cases. No abnormality in vitreous has also been reported by Buyukyildiz in two cases of retinal toxicity caused by 2 mg/0.1 ml cefuroxime intra-cameral injection [3]. The dose of cefuroxime injection was much higher in Delyfer et al. study [4] than the presented cases and Buyukyildiz et al. cases [3]. The different doses of cefuroxime injection during cataract surgery may lead to the different findings in vitreous.

Topical nonsteroidal anti-inflammatory drugs and corticosteroids have been reported to be effective and safe therapy for preventing post-surgical ocular inflammatory and macular edema [17–20]. Thus combination of nonsteroidal anti-inflammatory drugs and corticosteroids was applied in the present cases topically as routine anti-inflammation treatment after phacoemulsification. The SD-OCT image revealed a quick recovery from the macular edema without any special surgical intervention 1 week later. Delyfer et al. [4] also reported that retinal injury and visual dysfunction induced by intra-cameral excessive cefuroxime injection were able to recover to normal without surgery intervention after 6 weeks. The recovery time was shorter in the present cases than previous report. That may be due to the much lower concentration of cefuroxime solution used in the two cases. These results suggest that early macular edema with extensive serous retinal detachment which may be attributed to cefuroxime toxicity are restorable. Routine anti-inflammatory treatment is sufficient and do not require excessive interventions.

## Consent

Written informed consent was obtained from the patients for publication of this case report and any accompanying images.

## Abbreviations

SD-OCT: spectral domain optical coherence tomography
OCT: optical coherence tomography
IOL: intraocular lens
CME: cystoid macular edema
FA: fluorescein angiograms
RPE: retinal pigment epithelium
